# Clinical and genetic risk factors for new-onset diabetes mellitus after transplantation (NODAT) in major transplant centres in Malaysia

**DOI:** 10.1186/s12882-020-02052-9

**Published:** 2020-09-07

**Authors:** Rhanye Mac Guad, Andrew W. Taylor-Robinson, Yuan Seng Wu, Siew Hua Gan, Nur Lisa Zaharan, Roma Choudhury Basu, Constance Sat Lin Liew, Wan Ahmad Hafiz Wan Md Adnan

**Affiliations:** 1 Department of Biomedical Science and Therapeutics, Faculty of Medicine & Health Science, Universiti Malaysia Sabah, Jalan UMS, 88400 Kota Kinabalu, Sabah Malaysia; 2 Infectious Diseases Research Group, School of Medical & Applied Sciences, Central Queensland University, Rockhampton, QLD 4702 Australia; 3 Department of Biochemistry, Faculty of Medicine, Bioscience and Nursing, MAHSA University, Jenjarom, Selangor Darul Ehsan Malaysia; 4 School of Pharmacy, Monash University Malaysia, Subang Jaya, Selangor Darul Ehsan Malaysia; 5 Department of Pharmacology, Faculty of Medicine, Universiti Malaya, Kuala Lumpur, Malaysia; 6 Clinical Investigation Centre, University Malaya Medical Centre, Kuala Lumpur, Malaysia; 7 Medical Based Department, Faculty of Medicine & Health Science, Universiti Malaysia Sabah, Kota Kinabalu, Sabah Malaysia; 8 Division of Nephrology, Department of Medicine, Faculty of Medicine, Universiti Malaya, Kuala Lumpur, Malaysia

**Keywords:** New-onset diabetes, Post-renal transplant, Polymorphism, Risk factors, Cyclosporine, Immunosuppression

## Abstract

**Background:**

New-onset diabetes after transplantation (NODAT) is associated with reduced patient and graft survival. This study examined the clinical and selected genetic factors associated with NODAT among renal-transplanted Malaysian patients.

**Methods:**

This study included 168 non-diabetic patients (58% males, 69% of Chinese ethnicity) who received renal transplantation between 1st January 1994 to 31st December 2014, and were followed up in two major renal transplant centres in Malaysia. Fasting blood glucose levels were used to diagnose NODAT in patients who received renal transplantation within 1 year. Two single nucleotide polymorphisms (SNPs), namely; rs1494558 (interleukin-7 receptor, IL-7R) and rs2232365 (mannose-binding leptin-2, MBL2) were selected and genotyped using Sequenom MassArray platform. Cox proportional hazard regression analyses were used to examine the risk of developing NODAT according to the different demographics and clinical covariates, utilizing four time-points (one-month, three-months, six-months, one-year) post-transplant.

**Results:**

Seventeen per cent of patients (*n* = 29, 55% males, 69% Chinese) were found to have developed NODAT within one-year of renal transplantation based on their fasting blood glucose levels. NODAT patients had renal transplantation at an older age compared to non-NODAT (39.3 ± 13.4 vs 33.9 ± 11.8 years, *p* = 0.03). In multivariate analysis, renal-transplanted patients who received a higher daily dose of cyclosporine (mg) were associated with increased risk of NODAT (Hazard ratio (HR) =1.01 per mg increase in dose, 95% confidence interval (CI) 1.00–1.01, *p* = 0.002). Other demographic (gender, ethnicities, age at transplant) and clinical factors (primary kidney disease, type of donor, place of transplant, type of calcineurin inhibitors, duration of dialysis pre-transplant, BMI, creatinine levels, and daily doses of tacrolimus and prednisolone) were not found to be significantly associated with risk of NODAT. GA genotype of rs1494558 (HR = 3.15 95% CI 1.26, 7.86) and AG genotype of rs2232365 (HR = 2.57 95% CI 1.07, 6.18) were associated with increased risk of NODAT as compared to AA genotypes.

**Conclusion:**

The daily dose of cyclosporine and SNPs of IL-7R (rs1494558) and MBL2 (rs2232365) genes are significantly associated with the development of NODAT in the Malaysian renal transplant population.

## Background

The first successful renal transplant was performed between identical twins in Boston, U.S.A on 23rd of December 1954 [1]. Over the ensuing decades, tremendous improvements had been achieved in the practice and outcomes of renal transplantation, including increased rates of graft and patient survival [2]. Unsurprisingly, transplantation has been a preferred option for treating patients with end-stage renal disease (ESRD) as compared to other treatment modalities [3]. However, new-onset diabetes mellitus after transplantation (NODAT) is a known complication among renal transplant recipients [4] and remains a challenge. Thus, it is essential to identify the risk factors that predispose this subset of patients, with the aim of treating them early.

Chow et al. in their systematic reviews have identified various risk factors that could predispose to the development of NODAT, including the use of corticosteroids and calcineurin inhibitors [5]. Other patient factors that have been shown to predispose to NODAT are age, ethnicity, family history, and body weight [6]. On the other hand, a meta-analysis of genetic association studies has identified the possible association of single nucleotide polymorphisms (SNPs) with NODAT, such as Interleukin-7 (IL7) rs1494558, Potassium Voltage-Gated Channel Subfamily Q Member 1 (KCNQ1) rs2237892, and Transcription Factor 7 Like 2 (TCF7L2) rs7903146 [7], indicating that genetic factors play a part in NODAT development.

β-cell dysfunction is considered as the main contributing factor to the development of NODAT due to the alteration in insulin secretion [8]. Several cytokines have been identified to induce pancreatic β-cell inflammation. Genetic variations of interleukins; IL-7R, IL-17E, IL-17R, and IL-17RB, which was recently reported to be associated with type 1 diabetes mellitus, could be associated with the pathogenesis of NODAT in renal-transplanted patients [9]. Mannose-binding lectin 2 (MBL2), a major recognition molecule of the lectin pathway of complement activation [10] is a candidate gene that may play an important role in inflammatory damage [11] after organ transplantation.

The Malaysian National Transplant Registry reported an impressive outcome of renal transplantation in this country, with more than 90% of patients with graft survival at one-year post-renal transplantation [12]. Given the increasing prevalence of risk factors, such as type 2 diabetes mellitus and hypertension in Malaysia [13], a higher incidence of end-stage kidney disease requiring renal transplantation would be expected in the future. Two clinical studies conducted earlier showed that the incidence of NODAT in Malaysia ranges from 5.5% [14] to 13.3% [15] at one-year post-transplantation. However, both studies were performed in a single centre and did not include a genetic component. The role of genetic polymorphism in the development of NODAT in Malaysian renal-transplanted patients is still unknown.

This study aimed to examine the possible association between selected clinical parameters and genetic polymorphisms with the development of NODAT in Malaysian renal transplant patients. Two common SNPs in Asian, the IL-7 receptor (rs1494558) and MBL2 (rs2232365), will be examined in association with the development of NODAT in this population.

## Methods

### Study design and patient population

**Fig. 1:**
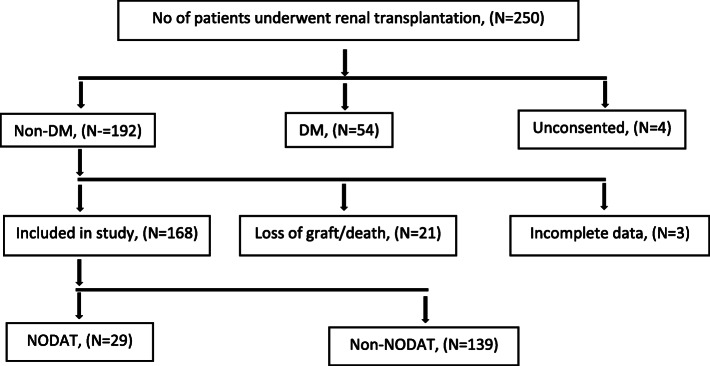
Schematic flow chart of participants recruitment

Malaysian patients who underwent renal transplant between 1st January 1994 to 31st December 2014 who were followed-up at two main renal transplant centres, namely Hospital Kuala Lumpur (HKL) and University of Malaya Medical Centre (UMMC) were identified (*n* = 250). Patients without a history of diabetes mellitus before renal transplant and had attended their follow-up at the two hospitals were recruited into the study (*n* = 192) (Fig. 1). This study was approved by the Medical Research and Ethics Committee of the Ministry of Health Malaysia (reference no NMMR-14-1527-21,690 (IIR) and conformed with the ethical regulations of the World Medical Association and the Declaration of Helsinki. Written informed consent was obtained from the patients before they participated in the study.

### Demographic and clinical information

Demographic information and clinical information were obtained retrospectively from medical records. Demographic information included gender, age and ethnicity while clinical parameters included primary kidney disease, duration of dialysis prior to transplant, place of transplant, age at transplant, types of the donor and immunosuppressive protocol. Retrospective data at one-month, three- months, six-months and one-year post-transplant were obtained for levels of fasting blood glucose (FBG) and creatinine, body mass indices (BMI), types of immunosuppressive agents and dosage of drugs. Patients without FBG data (*n* = 3), those with loss of graft or those who died within 1 year post-transplant were excluded (*n* = 21). All patients received a standard triple immunosuppressive maintenance therapy with calcineurin inhibitors (either cyclosporine or tacrolimus), mycophenolate mofetil (MMF) and corticosteroids (prednisolone) within 1 year of transplant. The patients who had switched from one calcineurin inhibitor to another were considered to have been exposed to both.

### Definition of new-onset diabetes mellitus after transplantation (NODAT)

NODAT was diagnosed according to the levels of FBG used by the American Diabetes Association Guideline: FBG ≥126 mg/dl (7.0 mmol/l) and not on oral hypoglycaemic agents or insulin at any point of follow up period within one-year of renal transplant [16, 17].

### Blood collection

Patients were invited to provide DNA samples during their routine clinic follow-up for this study. Five milliliter of whole blood was drawn into the EDTA tube and kept in an icebox before being transported to the laboratory for DNA extraction.

### DNA extraction and genotyping

DNA samples were extracted using the QIAamp Blood Purification kit (Qiagen, Hilden, Germany) as per manufacturer protocols. DNA was stored at − 80 °C until processed. Two SNPs, the IL7R (rs1494558) and MBL2 (rs2232365) were genotyped using the Sequenom Mass Array platform (Sequenom, USA). The procedure of genotyping is described elsewhere [18].

### Statistical analyse1s

A total of 168 patients with clinical and genotyping data were included in the final statistical analysis. Categorical variables were expressed as percentages, while continuous variables were expressed as mean with standard deviation (SD). Patients were categorized into NODAT NODAT (*n* = 29, 17%) and non-NODAT (*n* = 139, 83%). Comparisons of demographic and clinical variables between the two groups were performed using the chi-squared test for categorical variables and Student’s t-test or Mann-Whitney U test for continuous variables as appropriate.

The patients were followed-up using for time-points (one-month, three-months, six-months and one-year) post-transplant. Univariate and multivariate Cox proportional hazard regression analyses were used to examine the relationship between demographics and clinical covariates, and the development of NODAT utilizing the four time-points. NODAT was considered as the ‘event’ and patients were followed up until they developed NODAT, or until the end of the one-year study period for those who did not develop NODAT. Covariates were selected from the univariate analysis, considering a *p* < 0.05 as being statistically significant. The risks of NODAT with each parameter were presented as adjusted hazards ratio (HR) and 95% confidence intervals (CI).

## Results

### Patients’ characteristics

A total of 168 patients were included in this study (58% males, mean age at transplant: 34.8 ± 12.1 year), with 48% of patients (*n* = 80) were recruited from the UMMC while the remaining were from Hospital Kuala Lumpur (*n* = 88). Of those, 29 patients (17%) developed NODAT within one-year of post-renal transplant.

Under the ethnicity stratification, Chinese patients made up the majority of renal transplant recipient in this study (68%). Two-thirds of the patients underwent their renal transplant in Malaysia (*n* = 114) while others had their transplants performed overseas (*n* = 54) as presented in Table 1. The kidney donors were living-related (41%), living unrelated (11%) and cadaveric grafts (48%). These patients were administrated with either cyclosporine (54%) or tacrolimus (40%), while others received both immunosuppressant medications within a year of transplantation.

**Table 1:** Comparison of clinical characteristics between Malaysian renal transplant recipients who developed NODAT and those without NODAT at 1-year post- transplant

	All patients (*n* = 168) N (%) or mean ± SD	NODAT (*n* = 29) N (%) or mean ± SD	NON-NODAT (*n* = 139) N (%) or mean ± SD	*p*
Gender				0.70
- Male	98 (58)	16 (6)	82 (84)	
- Female	70 (42)	13 (19)	57 (81)	
Age at transplant	34.8 (12.1)	39.3 ± 13.4	33.9 ± 11.8	**0.03**
Ethnicities				0.84
- Malay	34 (20)	5 (15)	29 (85)	
- Chinese	115 (69)	20 (17)	95 (83)	
- Indian	19 (11)	4 (11)	15 (79)	
Primary kidney disease				0.20
- Glomerulonephritis	51 (30)	9 (18)	42 (82)	
- Hypertension	19 (11)	6 (32)	13 (68)	
- Others	98 (59)	14 (14)	84 (86)	
Type of donor				0.76
- Living related	71 (45)	11 (15)	60 (85)	
- Living non-related	20 (42)	3 (15)	17 (85)	
- Cadaveric	77 (12)	15 (20)	62 (80)	
Place of transplant				0.05
- Local	114 (67)	15 (13)	98 (87)	
- Overseas	54 (32)	14 (26)	40 (74)	
Types of calcineurin inhibitors				0.66
- Cyclosporine	90 (54)	15 (17)	75 (83)	
- Tacrolimus	67 (40)	11 (16)	56 (84)	
- Both	11 (6)	3 (27)	8 (73)	
Duration of dialysis (month)	33.0 (40.5)	23.1 ± 23.5	35.5 ± 43.3	0.09
BMI (kg/m^2^)	21.3 (4.6)	21.9 ± 4.6	21.1 ± 4.4	0.35
Levels of creatinine (μmol/L)	127 (122.6)	112.24 ± 34.2	130.11 ± 99.1	0.68
Fasting blood glucose (μmol/L)	5.5 (1.4)	6.5 ± 1.8	5.2 ± 0.7	< 0.0001
Average daily dose of cyclosporine (mg/kg)	295.4 (116.5)	328.8 ± 125.8	280.6 ± 92.3	0.15
Average daily dose of tacrolimus (mg/day)	7.5 (3.4)	7.5 ± 3.1	7.8 ± 2.4	0.55
Average daily dose of prednisolone (mg/day)	15.8 (4.4)	16.2 ± 2.6	15.8 ± 2.6	0.45

### Demographic and clinical variables associated with NODAT

Comparing those who developed NODAT and those who did not, patients with NODAT were significantly older at transplant (39.3 ± 13.4 vs 33.9 ± 11.8) (Table 1). There were no significant differences between NODAT and non-NODAT patients in terms of gender, ethnicities, primary kidney disease, type of donor, place of transplant and the duration of dialysis before transplant. Besides, there were no significant differences between the two groups in terms of their BMI, serum creatinine and average daily doses of immunosuppressants at one-year post-transplant.

A significantly increased risk of NODAT with increased age at transplant, patients of Chinese ethnicity compared to Malay ethnicity, and patients with hypertension as compared to those with glomerulonephritis was found in the univariate analysis (Table 2). The univariate analysis also showed that increased duration of dialysis prior to transplant was associated with a lower risk of NODAT. Also, patients prescribed higher doses of cyclosporine, tacrolimus and prednisolone were associated with a significantly increased risk of NODAT per unit mg increase in the dose of each drug.

**Table 2:** Risk of development of NODAT (unadjusted and adjusted) in Malaysian renal transplant recipients according to clinical characteristics presented as hazard ratio (HR) with 95% confidence of interval (CI)

Clinical characteristics	Unadjusted HR (95% CI), *p*	Adjusted^a^ HR (95% CI), *p*
Gender ^b^		
- Females	0.99 (0.65, 1.52), 0.98	0.70 (0.34, 1.43), 0.33
Age at transplant	1.04 (1.02, 1.06), < **0.0001**	1.01 (0.97, 1.06), 0.56
Ethnicities^c^		
- Chinese	2.27 (1.13, 4.54), **0.02**	1.46 (0.43, 4.96), 0.55
- Indian	2.19 (0.91, 5.27), 0.08	1.88 (0.47, 7.55), 0.37
Primary kidney disease^d^		
- Hypertension	1.89 (1.05, 3.39), **0.03**	1.10 (0.38, 3.21), 0.86
- Others	0.84 (0.58, 1.53), 0.82	0.77 (0.17, 3.55), 0.74
Type of donor^e^		
- Living related	1.07 (0.69, 1.65), 0.51	0.60 (0.17, 2.07), 0.59
- Living non-related	0.65 (0.29, 1.45), 0.29	0.58 (0.18, 1.9), 0.38
Place of transplant^f^		
- Overseas	1.42 (0.93, 2.17), 0.10	1.04 (0.42. 2.56), 0.93
Types of calcineurin inhibitors^g^		
- Tacrolimus	0.95 (0.61, 1.47), 0.81	0.95 (0.60, 1.51), 0.84
- Both	1.26 (0.54, 2.96), 0.59	0.89 (0.37, 2.12), 0.79
Duration of dialysis (month)	0.99 (0.90, 0.99), **0.04**	0.99 (0.98, 1.01), 0.27
BMI post-transplant	1.01 (0.97, 1.06), 0.44	0.93 (0.81, 1.06), 0.29
Levels of creatinine post-transplant	1.00 (0.99, 1.00), 0.58	1.00 (0.99, 1.02), 0.62
Average daily dose of cyclosporine	1.01 (1.00, 1.01), **< 0.0001**	1.01 (1.00, 1.01), **0.002**
Average daily dose of tacrolimus	1.15 (1.03, 1.27), **0.009**	1.10 (0.98, 1.23), 0.11
Average daily dose of prednisolone	1.11 (1.06, 1.16), **< 0.0001**	1.07 0.96, 1.20), 0.23

^a^Adjusted for age at transplant, ethnicities, primary kidney disease, duration of dialysis pre-transplant and doses of immunosuppressants

^b^ reference: Males ^c^ reference: Malay ethnicities

^d^ reference: Glomerulonephritis ^e^ reference: Cadaveric donor

^f^ reference: Transplanted in Malaysia ^g^ reference: cyclosporine as immunosuppressant

In the multivariate analysis, following adjustment for recipient’s age, gender, ethnicities, primary kidney disease, the duration of dialysis and dosage of immunosuppressants, the single parameter which remained significantly associated with a slightly higher risk of NODAT among the Malaysian renal transplant recipients is the daily dose of cyclosporine with HR of 1.01 (95% CI 1.00–1.01) per mg increase in dose.

### Genetic polymorphisms and incidence of NODAT

For rs1494558 of the IL7R variant, those with the GA genotype were associated with a significantly increased risk of NODAT in the pooled Malaysian renal transplant patients as compared to the AA genotype (adjusted HR = 3.15 (95% CI 1.26,7.86), as shown in Table 3. Meanwhile, the AG genotypes of the rs2232365 MBL2 variants were associated with a higher risk of NODAT with adjusted HR of 2.57 (95% CI 1.07, 6.18) compared to the AA genotype. The frequencies for rs1494558 and rs2232365 SNPs were not significantly different from those expected based on the Hardy–Weinberg equilibrium indicating that the sample was uniformly-distributed. The genotype frequencies for the rs1494558 and rs2232365 SNPs, according to ethnicity is presented in Table 4.

**Table 3:** Comparison of genotype frequencies for rs1494558 and rs2232365 and their risks of NODAT in Malaysian renal transplant recipients presented as HR and 95% CI

Genotype	NODAT (n, %)	Non NODAT (n, %)	Unadjusted HR (95% CI), *p*	Adjusted^a^ HR (95% CI), *p*
**rs1494558**				
- AA (*n* = 43)	6 (14%)	37 (86%)	ref	ref
- GA (*n* = 97)	18 (19%)	79 (81%)	1.98 (1.09, 3.61), 0.02	3.15 (1.26, 7.86), **0.01**
- GG (*n* = 27)	4 (15%)	23 (85%)	1.44 (0.66, 3.15), 0.36	1.22 (0.31, 4.91), 0.77
**rs2232365**				
- AA (*n* = 79)	12 (15%)	67 (85%)	ref	ref
- AG (*n* = 31)	5 (16%)	26 (84%)	0.86 (0.47, 1.57), 0.62	2.57 (1.07, 6.18), **0.04**
- GG (*n* = 57)	11 (11%)	46 (81%)	0.98 (0.61, 1.58), 0.95	0.80 (0.39, 1.66), 0.55

^a^Adjusted for ethnicities, age at transplant, primary kidney disease, duration of dialysis pre-transplant and average daily doses of immunosuppressants

**Table 4:** Comparison of genotype frequencies for rs1494558, rs2232365 according to ethnicities of Malaysian renal transplant recipients

Genotype	Pooled participants (n, %)	Chinese (n, %)	Malay (n, %)	Indian (n, %)	*p*
rs1494558					
- AA	43 (26%)	30 (26%)	9 (26%)	4 (21%)	0.98
- GA	97 (58%)	66 (58%)	19 (56%)	12 (63%)	
- GG	27 (16%)	18 (16%)	6 (18%)	3 (16%)	
rs2232365					
- AA	79 (47%)	58 (51%)	16 (47%)	5 (26%)	0.19
- AG	31 (18%)	18 (16%)	6 (18%)	7 (37%)	
- GG	57 (34%)	38 (33%)	12 (35%)	7 (37%)	

## Discussion

This study demonstrated that those with NODAT in the Malaysian population had a significantly higher age at transplant when compared to those without NODAT. In addition, a higher dose of cyclosporine appeared to confer a slightly higher risk for NODAT among transplanted patients per mg increase in dose. Polymorphisms in the IL7R (rs1494558) and MBL2 (rs2232365) genes were associated with a higher risk of NODAT among pooled Malaysian renal transplanted populations.

Despite similar diagnostic criteria, high variability in the incidence of NODAT within 1 year post-transplantation has been reported in different populations [19–21]. The incidence of NODAT at one-year post-transplantation in our study was slightly higher than the earlier reports by Foo et al. (13.3%) [15] and Lai et al. (5.5%) [14] in their studies in single centres in Malaysia. This finding indicates that variability could exist based on the socio-geographical location in this country. Nevertheless, our finding was consistent with that of our neighbouring county, Singapore, which consisted of a similar multi-ethnic population. Their incidence of NODAT was reported to be at 15.8% following one-year of transplantation in 2011 [6]. It should be noted that a vast majority of end-stage renal disease patients live in developing countries (such as South Africa and South East Asian countries) and could contribute to the increased number of transplanted patients and thus occurrence of NODAT worldwide [22].

Ageing has been consistently shown to be a significant risk factor for NODAT, especially among patients who are above 40 years of age [23]. This finding is not surprising considering the influence of age on the higher incidence of type 2 diabetes mellitus in the general population [24]. Despite our study population comprising of younger aged patients (mean age of 34.8 years old), those with NODAT were found to be older than those without (mean age 39.3 vs 33.9 years old). However, no significant increased risk was found in the multivariate model.

Several published reports had demonstrated a higher incidence of NODAT following the introduction of calcineurin inhibitors in renal transplantation [25]. In this study, we have not established a higher risk of NODAT with tacrolimus as compared to cyclosporine. Similarly, an earlier study among the Malaysian population by Lai et al. [14] found no significant difference in the risk of NODAT with the use of the two calcineurin inhibitors. This study found an increased risk of NODAT with increasing daily dose of cyclosporine (per unit mg increase). Although the risk is modest (HR = 1.01 (95% CI 1.00–1.01)) per mg increase in the daily dose, this is considered important, especially in those who are prescribed a high dosage of cyclosporine. Previous randomized control trial using different dosing regimen of cyclosporine has shown a higher incidence of post-transplant diabetes in patients receiving standard-dose cyclosporine (trough level 150–300 ng/ml), compared to lower-dose (50–100 ng/ml) [26].

Our study demonstrated a significant association between GA genotypes of rs1494558 in IL7R gene and NODAT risk when compared to the AA genotype. Beta-cell dysfunction is considered as the main contributing factor in the development of NODAT, leading to an alteration in insulin secretion [27]. One of the mechanisms of beta cells malfunction is increased expression of IL-7R, IL-17E, IL-17R and IL-17RB, and the association of these cytokines with NODAT among renal transplanted patients in several populations has been reported [27, 28]. In addition, we demonstrated that the AG heterozygous variant of the MBL2 gene (rs2232365) carries a higher risk of NODAT when compared with the AA variant. Since MBL plays an essential role in the lectin pathway of complement activation [10] and is therefore involved in the non-infectious inflammatory damage [11] including organ transplantation, genetic polymorphism of this molecule may alter insulin secretion from the pancreas.

In countries which are composed mainly of Caucasians, research findings have revealed the role of ethnicity in the development of NODAT [29]. Malay and Indian ethnicities constituted a smaller sample size as compared to the Chinese ethnicity in our study, as also reported by Lai et al. [14], a reflection of the demographics of our participating hospitals. After adjusting for possible confounders, there were no significant differences in the risk of NODAT based on ethnicity in this population. Consistent with the findings from Nepali R et al. [30], being males was not associated with a higher risk of NODAT compared to females in the current study.

Other studies have shown cadaveric kidney donor as having an increased risk of NODAT [31]. In our study, there was no significant increased risk of NODAT found in cadaveric donors compared to living transplant donors, probably due to a small number of cadaveric donors in this study. Although there were more patients with hypertensive who developed NODAT compared to those with glomerulonephritis, this associated was not significant after adjustment of covariates.

We did not establish any significantly increased risk of NODAT with increased BMI in this study. The mean BMI within 1 year of transplant for both groups in our study were within the normal range of BMI, suggesting that our renal transplanted patients were rather lean on average. As such, we were unable to capture the true risk of BMI in the development of NODAT in our transplanted population.

This study has several strengths. Two main transplant centres in the country were systematically evaluated for NODAT in the transplant patients according to the ADA guidelines, as previously recommended. Furthermore, patients were followed up longitudinally for 1 year (4-time points) based on the FBG as a criterion of NODAT, rather than using a single diagnostic point after one-year. To the best of our knowledge, this is the first study on the association of genetic polymorphism and NODAT (17%) among renal transplanted patients in Malaysia.

We could not exclude the possibility of selection bias, since patients who were transplanted in the study period who died, were transferred to other transplant centres, or did not attend follow-up sessions during the recruitment year, were excluded from the study. Despite the recommendation to use combined diagnostic criteria with glycosylated haemoglobin (HbA_1_C) for glucose measurements [32], HbA1c monitoring was not routinely performed in the hospitals setting during the study period chosen. The retrospective data obtained were not linked to pharmacy data, and the cumulative dose of immunosuppressants as taken by patients were not available. The dosages of the immunosuppressants were obtained using clinical records and not specified using the World Health Organization (WHO) criteria of defined daily dose (DDD). Thus, calculations of daily dose may not be truly reflective of doses received and taken by patients.

Additionally, the exclusion of patients with a history of type 2 diabetes mellitus may lead to the selective exclusion of risk alleles, which could lead to an underestimation of the true risk conferred by these genetic variants. Some clinical information was not available in the clinical records, such as the presence of a family history of diabetes mellitus. Therefore, a multi-centre prospective study in the future is recommended to provide more insight into the contributors of NODAT, especially the genetic associations of inflammatory markers gene.

## Conclusion

A potential measure to reduce diabetes risk after renal transplantation, including the choice of an optimal dose of cyclosporine during the first year of post-transplantation, should be taken into consideration to prevent NODAT. A comprehensive renal transplant registry that includes various clinical outcomes and drug information is needed for future well-designed studies on renal transplanted patients in Malaysia. Future studies to examine genetic polymorphisms, especially those involved in beta cell dysfunction, should be undertaken to explore their potential implication, as SNPs such as rs1494558 in IL7R and rs2232365 in MBL2 were shown to be associated with NODAT in this population.

## Data Availability

The datasets generated during and/or analyzed during the current study are not publicly available but are available from the corresponding author on reasonable request.

## Abbreviations

NODAT: New-onset diabetes mellitus after transplantation
SNP: Single nucleotide polymorphism
ESRD: End-stage renal disease
IL-7: Interleukin-7
KCNQ1: Potassium voltage-gated channel subfamily Q member 1
TCF7L2: Transcription factor 7 like 2
IL-7R: Interleukin-7R
IL-17E: Interleukin-17E
IL-17R: Interleukin-17R
IL-17RB: Interleukin-17RB
MBL2: Mannose-binding lectin 2
HKL: Hospital Kuala Lumpur
UMMC: University of Malaya Medical Centre
FBG: Fasting blood glucose
BMI: Body mass indices
MMF: Mycophenolate mofetil
CI: Confidence intervals
PKD: Polycystic kidney disease
HbA1C: Glycosylated haemoglobin
